# Increased prevalence of the CVD-associated *ANRIL* allele in the Roma/Gypsy population in comparison with the majority Czech population

**DOI:** 10.1590/1678-4685-GMB-2020-0405

**Published:** 2021-04-30

**Authors:** Jaroslav A. Hubáček, Lenka Šedová, Věra Olišarová, Věra Adámková, Valérie Tóthová

**Affiliations:** 1 Institute for Clinical and Experimental Medicine, Experimental Medicine Centre, Prague, Czech Republic.; 2 Charles University, 1st Faculty of Medicine, 3rd Department of Internal Medicine, Prague, Czech Republic.; 3 University of South Bohemia, Faculty of Health and Social Sciences, České Budějovice, Czech Republic.; 4 Institute for Clinical and Experimental Medicine, Department of Preventive Cardiology, Prague, Czech Republic.

**Keywords:** ANRIL, polymorphism, cardiovascular disease, Roma/Gypsy, ethnic differences

## Abstract

Cardiovascular disease (CVD) is a major cause of death around the world, with highest prevalence reported in minority Roma/Gypsy populations living in developed countries. Whether these differences are caused by unhealthy lifestyles or genetic factors remain unknown. The aim of our study was to examine the genotype frequencies of the rs10757274 polymorphism in the 9p.21 locus within *ANRIL* (antisense non-coding RNA in the INK4 locus), a long non-coding RNA located in the vicinity of the CDKN2A/2B inhibitors loci. *ANRIL* is understood to be the strongest genetic determinant of CVD in Caucasians. Using PCR-RFLP, we analysed the *ANRIL* rs10757274 polymorphism in 298 non-Roma (50% male) and 302 Roma/Gypsy (50% male) adult (39.5 ± 15.1 years and 39.2 ± 12.8 years, respectively) subjects. We found that frequencies of the *ANRIL* GG, GA and AA genotypes were 20.1%, 52.4% and 27.5% in the majority population and 32.9%, 47.9% and 19.2% in Roma/Gypsy subjects, respectively. The distribution of genotypes was deemed significantly different at P < 0.001. Within the Roma/Gypsy population, we detected increased prevalence of the CVD-associated GG genotype. Increased prevalence of CVD among Roma/Gypsies subjects may be significantly linked to genetic background.

Cardiovascular diseases and myocardial infarction (CVD, MI) are the major causes of death in industrial countries. It is widely acknowledged that, next to environmental and lifestyle risk factors (Bhatnagar, 2017), genetic predisposition plays an important role in CVD determination (Dainis and Ashley, 2018).

Although previous publications on this topic are sparse and typically characterised by low numbers of examined subjects and non-representative selections, they report that prevalence of CVD risk factors in Roma/Gypsy communities is higher than in majority populations (Dobranici *et al*., 2012). A Czech national survey (Antosova *et al*., 2014) reported that prevalence of heart disease in the Roma/Gypsy population living in the Czech Republic was more than double that of the majority population. The question remains whether increased prevalence of CVD is caused not just by unhealthy lifestyles but also by genetic factors, as genetic differences between Roma/Gypsy and non-Roma populations have been widely documented (Mydlárová Blaščáková *et al*., 2017; Hubacek *et al*., 2017, 2020; Nagy *et al*., 2017; Dlouhá *et al*., 2020).

A number of genome-wide association studies (GWAS) have detected a comprehensive list of common polymorphisms associated with increased risk of MI (for a summary, see Erdmann *et al*., 2018). Among the strongest are the single-nucleotide polymorphisms (rs10757274, rs10965215, rs1333040 and rs4977574 are among the most commonly studied) within *ANRIL* (antisense non-coding RNA in the INK4 locus; OMIM acc. N. 613149). A long non-coding regulatory RNA located in the 9p.21 locus (Helgadottir *et al*., 2007), *ANRIL* is clustered within the cyclin-dependent kinase inhibitors CDKN2A and CDKN2B. *ANRIL* is understood to regulate - through a complex mechanism - a number of processes implicated in endothelial injury and subsequent development of atherosclerosis. That *ANRIL* is expressed in almost all human tissue highlights the extraordinary importance of this regulatory RNA.

At-risk *ANRIL* alleles are associated with an approximate 30-35% increased risk of MI per each allele (Palomaki *et al*., 2010). Interestingly, despite the homogeneous and strong association between *ANRIL* SNPs and CVD risk among different ethnicities (Hu *et al*., 2019), identical variants are not typically associated with traditional CVD risk factors such as smoking, dyslipidaemia, obesity and hypertension (with the exception of diabetes). Nevertheless, even after adjusting for diabetes, the highly significant association between *ANRIL* polymorphisms and CVD indicates the independent influence of this locus on these two major non-communicable diseases.

Two groups of adult populations (the non-Roma Czech majority and the Roma/Gypsy ethnic minority) inhabiting a Czech region South Bohemia (Table 1; Hubacek *et al*., 2020) have been included in the study. Snowball sampling (Hughes *et al*., 1995) has been used to the Czech Roma/Gypsy subpopulation (N = 302) selection and quota sampling (Walter, 1989) for recruiting Czech Caucasians/Slavs (N = 298). All subjects were older than 18 years at the time of data and samples collection and theirs ethnicity was based on self-reported information (Adámková *et al*., 2015, Šedová *et al*., 2015, Hubáček **et al**., 2018).

Ethics committee at South-Bohemia University approved the study protocol and all participants signed the informed consent with participation in the study.

**Table 1 -:** General characteristics of examined subjects (data identifying cohorts have been published in Hubacek *et al*., 2020).

	Roma/Gypsies	Majority Slavs	P
N	302	298	
Age (years)	39.2 ± 12.8	39.5 ± 15.1	n.s.
% of females	50	50	n.s.
BMI (kg/m²)	29.9 ± 5.6	25.0 ± 6.0	0.01
SBP (mm Hg)	124.3 ± 20.9	124.9 ± 14.4	n.s.
DBP (mm Hg)	76.6 ± 12.7	77.2 ±10.6	n.s.
Total cholesterol (mmol/L)	5.1 ± 1.4	5.1 ± 1.1	n.s.
Glycaemia (>6 mmol/L) (%)	58.6	23.3	0.00001
Total body fat (>25%) (%)	69.4	61.7	0.05

DNA were isolated (“Xtreme DNA Isolation Kit”), from buccal cells collected through “DNA buccal swabs” (both Isohelix, Cell Projects Ltd, UK) according to manufacturer conditions.

The *ANRIL* rs10757274 genotype was analysed using the polymerase chain reaction-restriction fragment length polymorphism method, as described in detail elsewhere (Hubacek *et al*., 2016). Restriction fragments were separated using a 10% polyacrylamide gel. Fermentas International Inc. (Burlington, Ontario, Canada) provided all PCR chemicals, with PCRs performed on the MJ Research DYAD Disciple PCR device.

Deviance of genotype frequencies among the groups examined was analysed using the Hardy-Weinberg equilibrium principle (Court, 2005-2008). Differences in allele and genotype frequencies were compared using an online chi-square test (Stangroom). Comparisons were performed for dominant, co-dominant and recessive models.

The call rates achieved for the *ANRIL* rs10757274 SNP were 96.6% in the Czech majority population and 96.7% in the minority population. No significant gender differences in genotype frequencies were observed in either ethnic group (not presented in detail). Distributions of genotypes were in agreement with the Hardy-Weinberg equilibrium (P = 0.36 for the majority population and P = 0.70 for the minority population).

Within the minority Roma/Gypsy population, there were significantly more carriers of the CVD-associated GG genotype (P < 0.001; for further details and comparisons, see Table 2). Consequently, prevalence of the G allele was also significantly higher (56.8% vs. 46.4%; P < 0.0005).

**Table 2 -:** Distribution of *ANRIL* rs10757274 genotypes in the majority Czech non-Roma and minority Roma/Gypsy populations.

*ANRIL*	Czech majority		Roma/Gypsy minority		P
rs10757274	N	%	N	%	
GG	58	20.1	96	32.9	*0.0005
GA	151	52.4	140	47.9	^#^0.001
AA	79	27.5	56	19.2	^§^0.02
G	267	46.4	332	56.8	0.0005
A	309	53.6	252	43.2	

P-values are given for *GG vs. +A, ^#^GG vs. GA vs. AA, and ^§^+G vs. AA comparisons.

Despite the relatively low numbers of subjects enrolled in this study, the genotype frequencies observed for the majority population are in agreement with the frequency (22% of *ANRIL* rs10757274 GG homozygosity) reported in our previous study (Hubacek *et al*., 2016), as well as in neighbouring German population (Scheffold *et al*., 2011). In contrast, detected frequencies of the G allele in the minority Roma/Gypsy population (57%); are among the highest in the world and even higher than in the Indian population (Kumar *et al*., 2011), which is understood to be the origin of the settled Roma/Gypsy populations in Europe (Mendizabal *et al*., 2011).

Although examining a sole SNP is evidently insufficient at drawing major conclusions, we speculate that the increased prevalence of at-risk *ANRIL* genotypes in the Roma/Gypsy population could be an important modifier associated with higher prevalence of CVD in this ethnic group. To extend our findings, a further case-control study involving CVD-focused tagging of *ANRIL* SNPs within this ethnic group is required.

We conclude that CVD-associated *ANRIL* genotypes are more common in the Roma/Gypsy population compared to the majority Caucasian population.
